# Success rates in isolating mesenchymal stem cells from permanent and deciduous teeth

**DOI:** 10.1038/s41598-019-53265-4

**Published:** 2019-11-14

**Authors:** Kengo Nakajima, Ryo Kunimatsu, Kazuyo Ando, Tomoka Hiraki, Kodai Rikitake, Yuji Tsuka, Takaharu Abe, Kotaro Tanimoto

**Affiliations:** Department of Orthodontics and Craniofacial Developmental Biology, Division of Dental Sciences, Biomedical Sciences Major, Hiroshima University Graduate School of Biomedical & Health Sciences, 1-2-3 Kasumi, Minami-ku, Hiroshima, 734-8553 Japan

**Keywords:** Cell growth, Craniofacial orthodontics

## Abstract

Stem cells from human exfoliated deciduous teeth (SHED) and human dental pulp stem cells (hDPSCs) have emerged as attractive cell sources for bone regeneration. However, the specific teeth and the conditions most suitable for stem cell isolation remain unclear. Therefore, the success rate of SHED and hDPSCs isolation, the patient age and remaining root length in deciduous teeth were evaluated. Successful isolation was defined as when the cell culture was maintained up to the third passage without any contamination or other issues. Remaining tooth length was calculated using the root-to-crown ratio from patient X-rays and compared to the norm value from the literature. The overall successful isolation rate of SHED and hDPSCs was 82% and 70%. The average patient ages at extraction of the deciduous teeth and permanent teeth were 11 years and 9 months, and 22 years and 10 months respectively. In the successful SHED group, the average remaining root length of the anterior deciduous teeth was 71.4%, and that of the deciduous molars was 61.4%. Successful isolation appears to be associated with patient age, length of the remaining root, and also mechanical stress and other factors. Tooth selection criteria need to be identified to improve the success rate.

## Introduction

Mesenchymal stem cells (MSCs) have emerged as a promising tool for tissue regeneration. Since the isolation of bone marrow MSCs (BMMSCs), MSC-like cells have been continuously discovered from various tissues. Human dental pulp stem cells (hDPSCs) were first isolated in 2000^1^, followed by isolation of stem cells from human exfoliated deciduous teeth (SHED) in 2003^2^. Subsequently, stem cells from the periodontal ligament and apical papilla were isolated and characterised^3,4^. SHED are derived from the pulp of deciduous teeth, which are clinically and biologically discarded tissues. Thus, among these MSC sources of the dental tissue, SHED represent the most accessible and promising cell source for tissue regeneration.

We previously reported successful bone regeneration using autogenous BMMSCs in a dog model of artificial alveolar cleft^5,6^. However, since bone marrow collection is an invasive procedure for the patient, we have focused on the bone regeneration potential of SHED and hDPSCs both *in vitro* and *in vivo*. Indeed, the *in vivo* transplantation of human SHED and hDPSCs for bone regeneration has been reported, similar to human BMMSCs (hBMMSCs)^7^. *In vitro*, SHED and hDPSCs show high proliferation activity, and have similar differentiation ability to osteoblasts as that of hBMMSCs^8^. Moreover, SHED have been applied for regeneration of mineralised tissue^9–13^. Thus, SHED and hDPSCs could be ideal tools for bone regeneration. However, the tooth source that is most suitable for the isolation of MSCs and ultimate bone regeneration remains to be determined. Accordingly, in this preliminary study, we evaluated the success rate of SHED and hDPSCs isolation from deciduous and permanent teeth and related this rate to the condition of the teeth and general patient characteristics.

## Methods

### Cell isolation and culture

Human dental pulp tissue from both permanent and deciduous teeth was obtained from clinically healthy patients who required extraction for orthodontic treatment at Hiroshima University Hospital. The collection of tissues and isolation of SHED and hDPSCs from patients were approved by the preliminary review board of the Epidemiological Research Committee of Hiroshima University (approval number: E-20-1). hDPSCs were isolated and cultured as previously described^1,2^. In brief, the extracted permanent teeth were split using bone forceps at the cementum-enamel junction after the periodontal tissue was removed. The obtained tissue from the dental chamber was minced by a scalpel in a mixture of 3 mg/mL type I collagenase (Gibco/Invitrogen, Carlsbad, CA, USA) and 4 mg/mL dispase (Godo Shusei Co., Ltd., Tokyo, Japan). The minced dental pulp tissue was then digested in the mixture at 37 °C for 30–60 min. Once digested, the solution was filtered through a 70-μm cell strainer (Falcon; BD Labware, Franklin Lakes, NC, USA) and centrifuged (1500 rpm, 10 min). The released cells were plated in a 35-mm dish containing alpha modified Eagle’s medium (Gibco/Invitrogen, Carlsbad, CA, USA) supplemented with 20% foetal bovine serum (FBS; Biological Industries, CT, USA), 100 U/mL penicillin (Meiji Seika Pharma Co., Ltd., Tokyo, Japan), 100 μg/mL kanamycin (Meiji Seika Pharma Co., Ltd., Tokyo, Japan), and 0.25 μg/mL amphotericin (MP Biomedicals, LLC., France), and incubated at 37 °C in a 5% CO_2_ incubator. The SHED isolation procedure is almost same as for hDPSCs, except that we rarely used forceps to split the deciduous teeth and we additionally used a 70 µm cell strainer to filter the solution after digestion.

Information on the patients’ age, gender, and extracted tooth types was obtained from medical records and X-rays after receiving written informed consent.

### Isolation success rate of SHED and hDPSCs

Successful isolation was defined when the cell culture reached the third passage (P3) without any contamination or other issues. Cells were observed under an optical microscope at the same time every day as on the day of plating, and the first confirmation day of stem cells was recorded. Confirmation day was defined that the day when cell adhesion was observed and colonies were formed.

### Calculation of remaining root length after physiological root absorption of SHED at extraction

The remaining root length was calculated using the root-crown ratio determined from the X-ray image taken just before extraction. The norm root-crown ratio of primary teeth was obtained from data reported by Black^14^, and then the percentage of remaining root length after physiological root absorption was calculated by dividing the patient’s root-crown ratio by the norm root-crown ratio. The variation of root length ratio according to success and failure of stem cell isolation was determined by grouping the teeth according to blocks of 20% variation (0–20%, 20–40%, 40–60%, 60–80%, and 80–100%).

### Statistical analysis

The Mann–Whitney U test was performed to compare the age at extraction and the period from plated day until the day of confirmation of cells between the success and failure groups; p < 0.05 and p < 0.01 were considered statistically significant.

### Ethical approval

All procedures performed in studies involving human participants were in accordance with the ethical standards of the institutional research committee and with the 1964 Helsinki Declaration and its later amendments or comparable ethical standards.

### Informed consent

Informed consent was obtained from all individual participants included in the study.

## Results

### Isolation success rates of SHED and hDPSCs

Isolation of SHED was successful in 18 of 22 extracted deciduous teeth (82%), which were cultured up to P3. In addition, hDPSCs were successfully isolated from 14 of the 20 permanent teeth (70%).

Both SHED and hDPSCs adhered to the cell culture dishes after floating from the minced tissue, and their isolation was confirmed by optical microscope observation within the first few days of culture up to approximately one week. Thereafter, multiple colonies were observed, and the cells were passaged after a sufficient increase. Three of the teeth in which SHED isolation failed were ultimately discarded because cell adhesion could not be confirmed even 21 days after seeding. One of the failure teeth was discarded just after plating because of contamination. In all six permanent teeth in which stem cells were not isolated, no cell adhesion was observed under the microscope.

### Success of SHED and hDPSCs isolation according to tooth type

Overall, cell isolation was performed from six deciduous incisors, ten deciduous canines, and six deciduous molars (Table 1). Although the total success rate of SHED was 82%, the success rate in the deciduous molars was lower (deciduous incisors: 72.4%, deciduous molars: 61.4%), which was likely due to the higher risk of contamination. However, isolation was successful in the three remaining deciduous molars.

**Table 1:** Cell isolation was performed from six deciduous incisors, ten deciduous canines, and six deciduous molars.

Subject	Sex	Age	Tooth type	Isolation
1	M	8y8m	64	s
2	M	11y2m	53	s
3	M	9y4m	65	s
4	M	7y2m	52	s
5	F	11y3m	62	s
6	F	8y0m	43	f
7	F	11y3m	53	s
8	M	13y1m	45	s
9	F	12y2m	53	s
10	M	21y1m	75	f
11	F	10y8m	53	s
12	F	17y6m	42	s
13	F	17y6m	62	s
14	F	12y0m	65	s
15	F	9y1m	63	s
16	M	8y1m	81	f
17	F	9y1m	53	s
18	F	15y1m	53	s
19	M	12y5m	63	s
20	M	12y5m	65	s
21	M	12y5m	63	f
22	F	9y8m	63	s

The two-digit system was used for tooth numbering. “Isolation” refers to the result of the isolation, with “s” meaning success and “f” meaning failure.

Isolation of hDPSCs was performed from two canines, twelve premolars, and six wisdom teeth (Table 2). Although the overall success rate was 70%, success was achieved for both canines (100%) that were extracted owing to root resorption. Premolars are frequently extracted for orthodontic treatment to align the teeth. Indeed, half of all permanent teeth in this study were premolars, and the success rate of hDPSCs isolation was 41.6%. Wisdom teeth are also frequently extracted after orthodontic treatment owing to caries and horizontal impaction. Of the six wisdom teeth extracted, hDPSCs isolation was successful in five of the six teeth that were cultured. The chamber of the extracted wisdom teeth is comparatively wider and thus a greater amount of dental pulp tissue was obtained compared to the other teeth.

**Table 2:** Isolation of hDPSCs was performed from two canines, twelve premolars, and six wisdom teeth.

Subject	Sex	Age	Tooth type	Isolation
1	F	13y8m	24	f
2	F	13y8m	45	f
3	F	25y0m	18	f
4	F	25y0m	28	s
5	F	17y1m	28	s
6	F	32y8m	23	s
7	F	25y4m	18	s
8	F	32y9m	14	s
9	F	32y9m	23	s
10	F	13y11m	14	f
11	F	21y8m	14	f
12	F	21y8m	24	f
13	F	20y0m	24	s
14	F	20y0m	14	s
15	F	34y4m	28	s
16	F	15y2m	18	s
17	F	20y7m	14	s
18	F	20y7m	34	s
19	M	26y7m	44	s
20	M	26y7m	14	s

The two-digit system was used for tooth numbering. “Isolation” refers to the result of the isolation, with “s” meaning success and “f” meaning failure.

### Success of SHED and hDPSCs isolation according to patient age at tooth extraction

**Figure 1:**
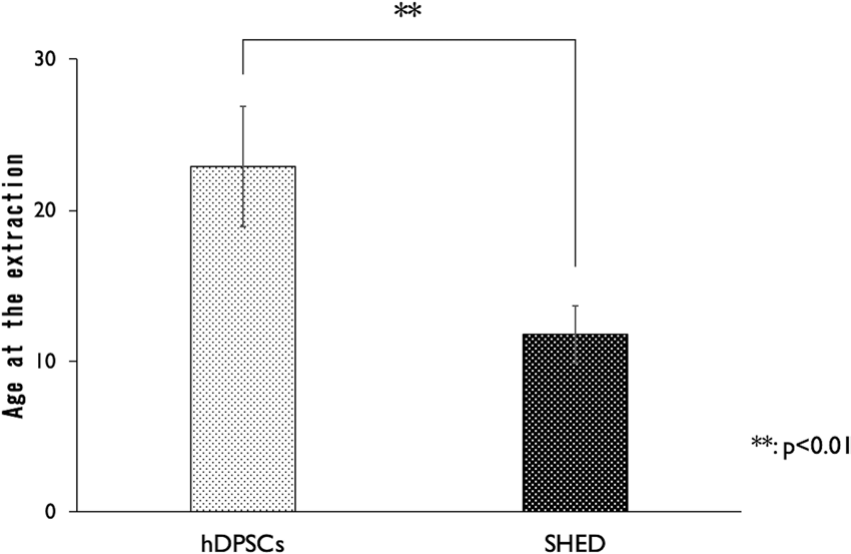
The average patient age in the SHED isolation group was 11 years and 9 months, and that of the hDPSCs isolation group was 22 years and 10 months, representing a statistically significant difference (p < 0.01).

**Figure 2:**
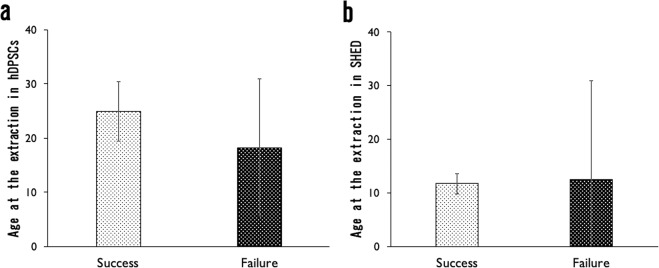
There was no significant difference between SHED success and failure groups with the Mann–Whitney U test (**a**). However, there was no influence of age on the success or failure of hDPSCs isolation (**b**).

The average patient age in the SHED isolation group was 11 years and 9 months, and that of the hDPSCs isolation group was 22 years and 10 months, representing a statistically significant difference (Fig. 1). In addition, there was no significant difference between the SHED success and failure groups with the Mann–Whitney U test. The average age for the success group was 11 years and 9 months, and for the failure group it was 12 years and 5 months (Fig. 2a). However, there was no influence of age on the success and failure of hDPSC isolation (Fig. 2b). The average age for the success group in hDPSCs isolation was 24 years and 11 months, and for the failure group, it was 18 years 3 months.

### Success of SHED and hDPSCs isolation according to interval from plating until confirmation

**Figure 3:**
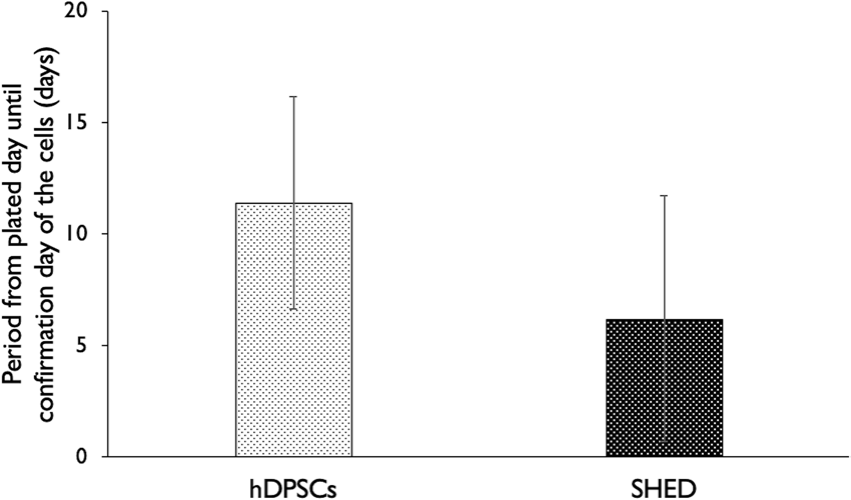
The average intervals from plating day until the confirmation day of SHED and hDPSCs were 7 and 11 days respectively, representing no significant difference.

The average intervals from plating day until confirmation day of SHED and hDPSCs were 7 and 11 days, respectively, representing no significant difference (Fig. 3).

### Remaining root length after physiological root absorption of SHED at extraction

**Figure 4:**
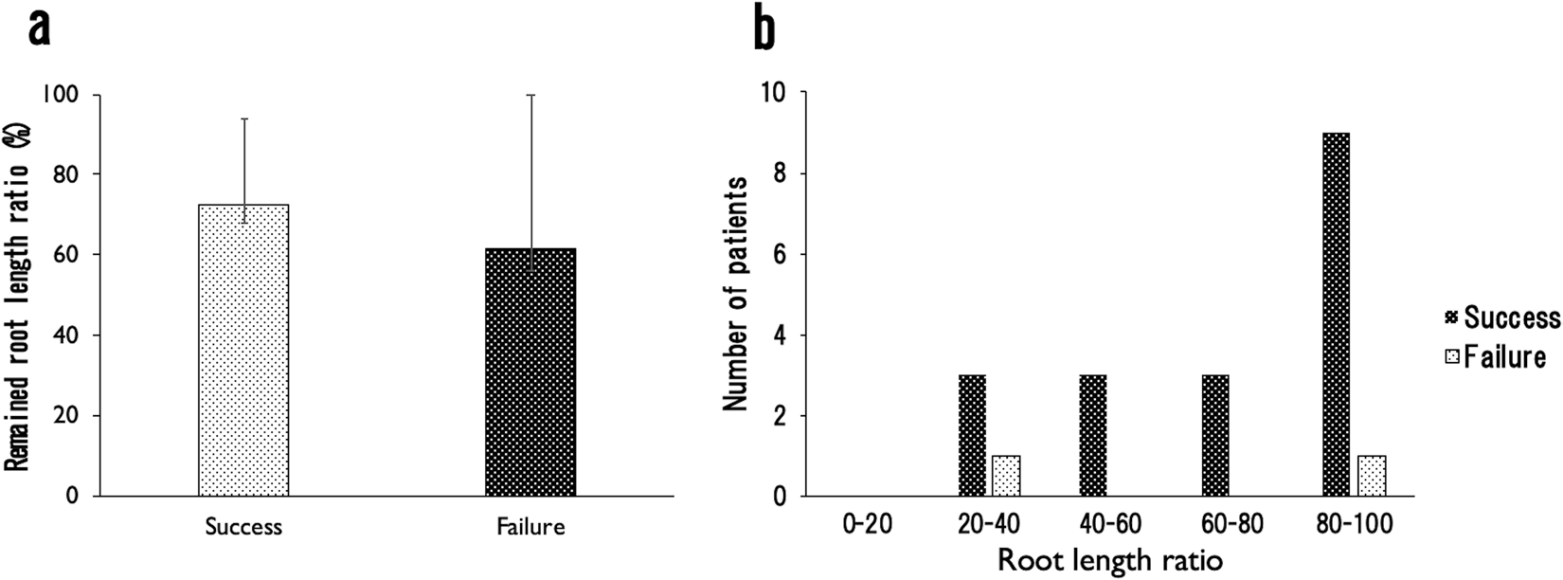
The average remaining root length of the deciduous teeth in the success group and the failure group was 65.4% and 60.5%. (**a**) Analysis of the variation of root length ratio showed that three patients with failure of SHED isolation were in the 20–40% group and X were in the 80–100% group. (**b**) In the success group, nine patients were in the 80–100% group with a total of only three in the other groups.

The remaining root length after physiological root absorption was calculated from the root-crown ratio of the deciduous teeth according to Black^14^. Three indistinct X-rays were excluded from analysis. There were 13 anterior deciduous teeth in the success group, with an average remaining root length of 72.4%. There were three deciduous molars in the success group, with an average remaining root length of 61.4% (Fig. 4a). In the failure group, one was a lower anterior deciduous tooth, and the other was an upper first deciduous molar. The root length of the former was 92.4%. However, the tooth showed heavy attrition and the permanent successor was a congenital missing tooth. The age of this patient was 21 years and 1 month. Moreover, the root chamber was much narrower than that of the others. The root length of the other teeth that failed in SHED isolation were 28.6% and 37.7%. However, contamination was observed during cell culture, and cell attachment could not be observed. Analysis of the variation of root length ratio showed that three patients with failure of SHED isolation were in the 20–40% group and X were in the 80–100% group (Fig. 4b). In the success group, nine patients were in the 80–100% group, with a total of only three in the other groups.

## Discussion

MSCs isolation has been attempted from various tissues to date, including the bone marrow^15^, adipose tissue (ADSCs)^16,17^, umbilical cord blood (UCB-MSCs)^18,19^, and periodontal tissue. After hDPSCs isolation^1^ and SHED isolation^2^, isolation of stem cells from the apical papilla^4,20^ and periodontal ligament^21^ was achieved. The success rate of the isolation of these cells is essential for their clinical applications. The success rate of BMMSCs and ADSCs was reported to be 100%^22^, whereas the success rate of UCB-MSCs is only 29%. However, this could be increased by 63% by precoating the culture dish with foetal calf serum taking into account only units of optical quality^23^. The success rate of MSC isolation from cryopreserved UCB is below 57%^24^. However, there are few reports related to the isolation rate of hDPSCs. Eubanks *et al*.^25^ reported that the success rate of hDPSCs isolation using FBS was 89%, and Lee *et al*.^26^ reported that cryopreservation of teeth negatively affected the isolation of hDPSCs, with a success rate of only 73%. Similarly, we found a 70% success rate of hDPSCs using FBS. However, this is the first report of the SHED isolation rate by using FBS, demonstrating improved success at 81.8%. Although these rates are relatively high, clinical application requires a rate as close to 100% as possible. The success rate of SHED may depend on which tooth is selected. Selection criteria need to be identified to improve the success rate.

Moreover, serum-free medium (SFM) or autoserum is needed to isolate the MSCs because long-term culture using medium containing FBS may result in the differentiation and malignant transformation of the cells. In addition, the use of FBS is associated with increased risks of infections of prions and pathogenic viruses. Therefore, the use of SFM is strongly recommended for clinical applications^27–30^. However, Karbanove *et al*.^31^ reported that SFM decreased the proliferative and differentiation ability of hDPSCs compared to culture with FBS. By contrast, Hirata *et al*.^30^ reported no significant difference in the survival rate of SHED culture with and without SFM, although SFM increased the proliferation ability. In any case, there is no doubt that more reliable research without using FBS or SFM is needed in the near future.

In our study, the patients in the SHED group were clearly younger than those in the hDPSCs group. This difference is expected because the reason for extraction in the SHED group was the eruption of permanent teeth and prolonged retention of deciduous teeth, whereas the teeth in the hDPSCs group were extracted for orthodontic treatments such as for the extraction of premolars and wisdom teeth. There was no significant difference in the success of isolation from both permanent and deciduous teeth. In SHED, because the success rate of cell isolation was high, the sample size of the failure group was very small. Therefore, despite significance using the Mann–Whitney U test, the results may not be statistically reliable. Furthermore, according to the condition of the teeth in the failure group, not only the age of patients when teeth were obtained, but also mechanical stress and other factors, might be associated with the success rate of the isolation.

The isolation of SHED was confirmed at a significantly earlier time during culture than that of hDPSCs. However, the standard deviation of the period from plating to confirmation was 4.9 days in hDPSCs and 5.5 days in SHED, indicating substantial individual variation.

To our knowledge, this is also the first report on the effect of the remaining root length ratio after physiological root absorption at the time of harvesting cells on the isolation success. In this study, the morphology of the tooth root and the number of roots of the tooth differed between the anterior teeth and the molar teeth. Therefore, these teeth were analysed separately. Furthermore, since the panoramic radiographs were not taken just before the extraction for some of the patients, the degree of absorption of the tooth root was calculated based on the norm values reported by Black *et al*.^14^. The remaining root length ratio of the successful group of anterior primary teeth was 72.4% (n = 13). However, this included one case in which the remaining root length ratios were only 30.1% and 43.2%, which could have influenced this result. Although the root length ratio of molars was only 61.4%, there were only four such teeth included in this group. However, the shortest root length ratio observed in this study was in a molar (28.6%) for which stem cell isolation failed, while another molar showed the greatest root length ratio overall (92.4%), although the following permanent tooth was a congenital missing tooth. Furthermore, since attrition was observed, and the patient was 21 years old, there was a possibility that the tooth had undergone long-term mechanical stress. Moreover, at the time of obtaining of the pulp tissue, stenosis of the root canal was observed, and the amount of collected pulp tissue was also very small. These facts suggest that the teeth most suitable for SHED isolation are those with only mild progression of root absorption and without mechanical stimulation. In addition, the SHED success rate is also strongly related to the root condition of the obtained deciduous teeth. Furthermore, it is necessary to consider not only the length of the remaining root but also the mechanical stress to the teeth, among other factors.

Accumulating evidence points to the potential of dental pulp as an accessible and promising cell source for bone regeneration^7–13^. In the near future, SHED and hDPSCs may become some of the best candidates for tissue regeneration. Therefore, more extensive studies and data on optimal methods for obtaining these cells will be of great importance to provide a safe and reliable regenerative medicine strategy.
